# Supplementary figures and images for: Increased Collagen Turnover Impairs Tendon Microstructure and Stability in Integrin α2β1-Deficient Mice
Source: Int J Mol Sci. 2020 Apr 18;21(8):2835. doi: 10.3390/ijms21082835 (PMC7215526; doi:10.3390/ijms21082835)

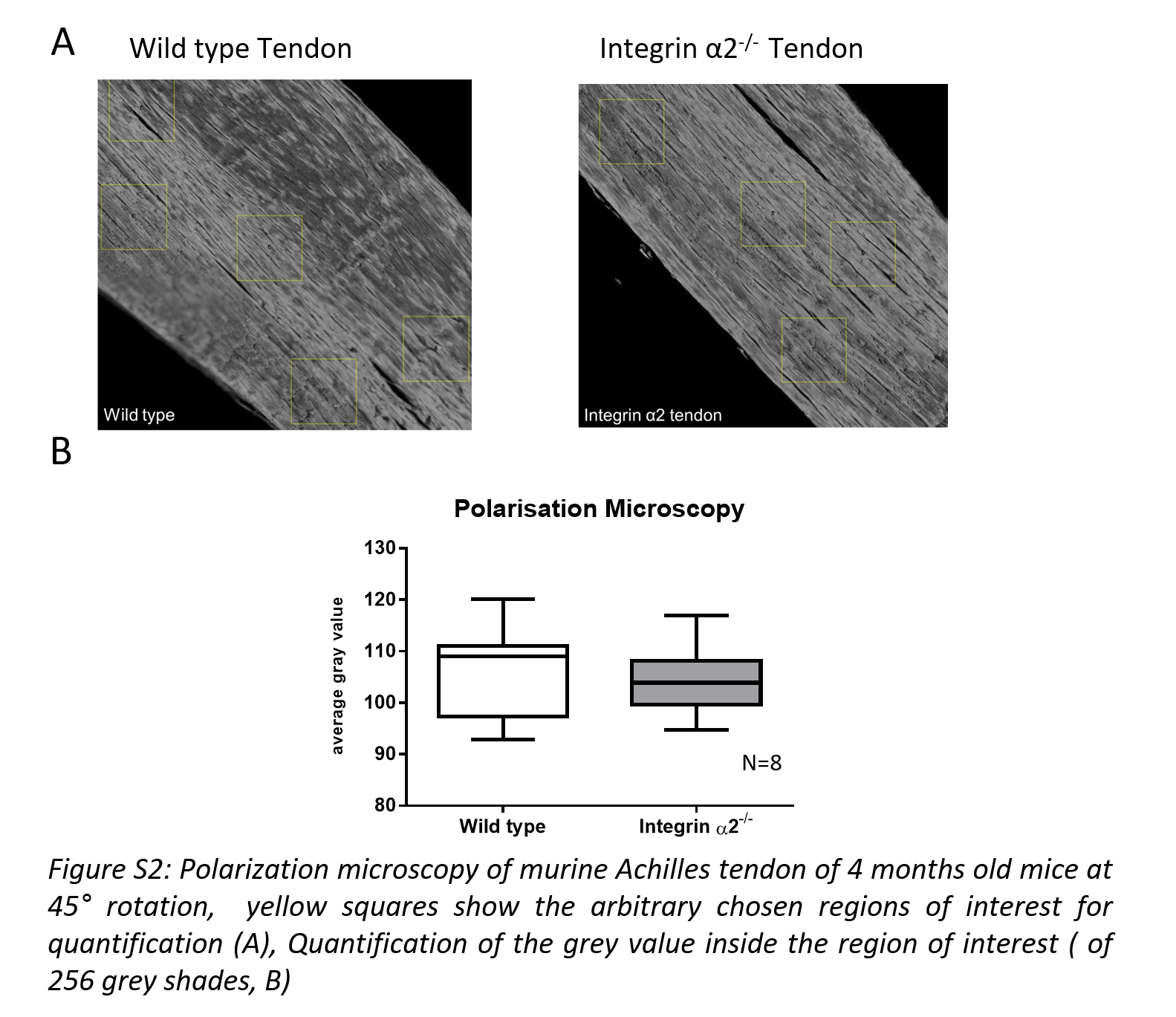

Supplement: Supplementary file 1 [file ijms-21-02835-s001.zip › Fig S2.tif]

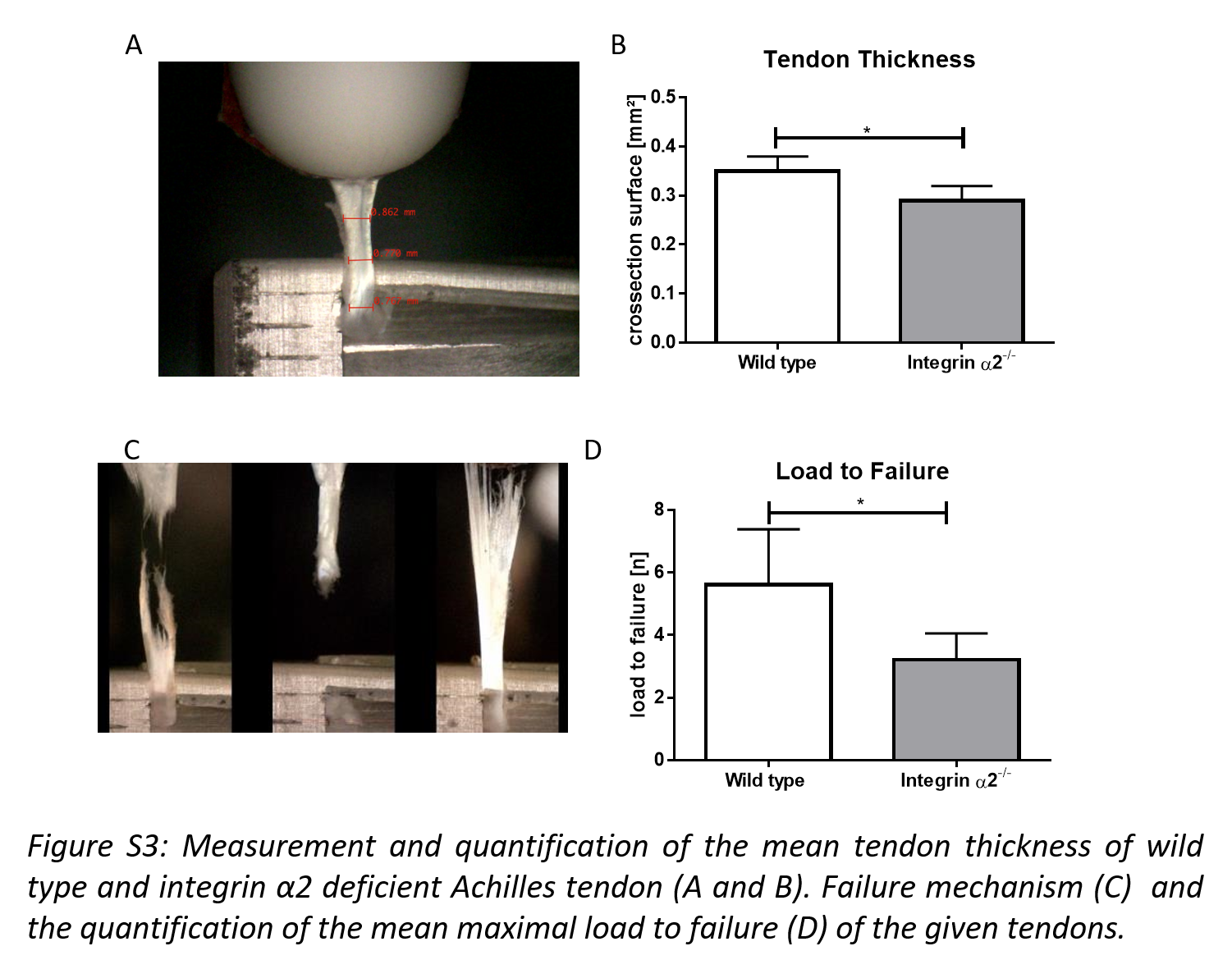

Supplement: Supplementary file 1 [file ijms-21-02835-s001.zip › Fig S3.tif]

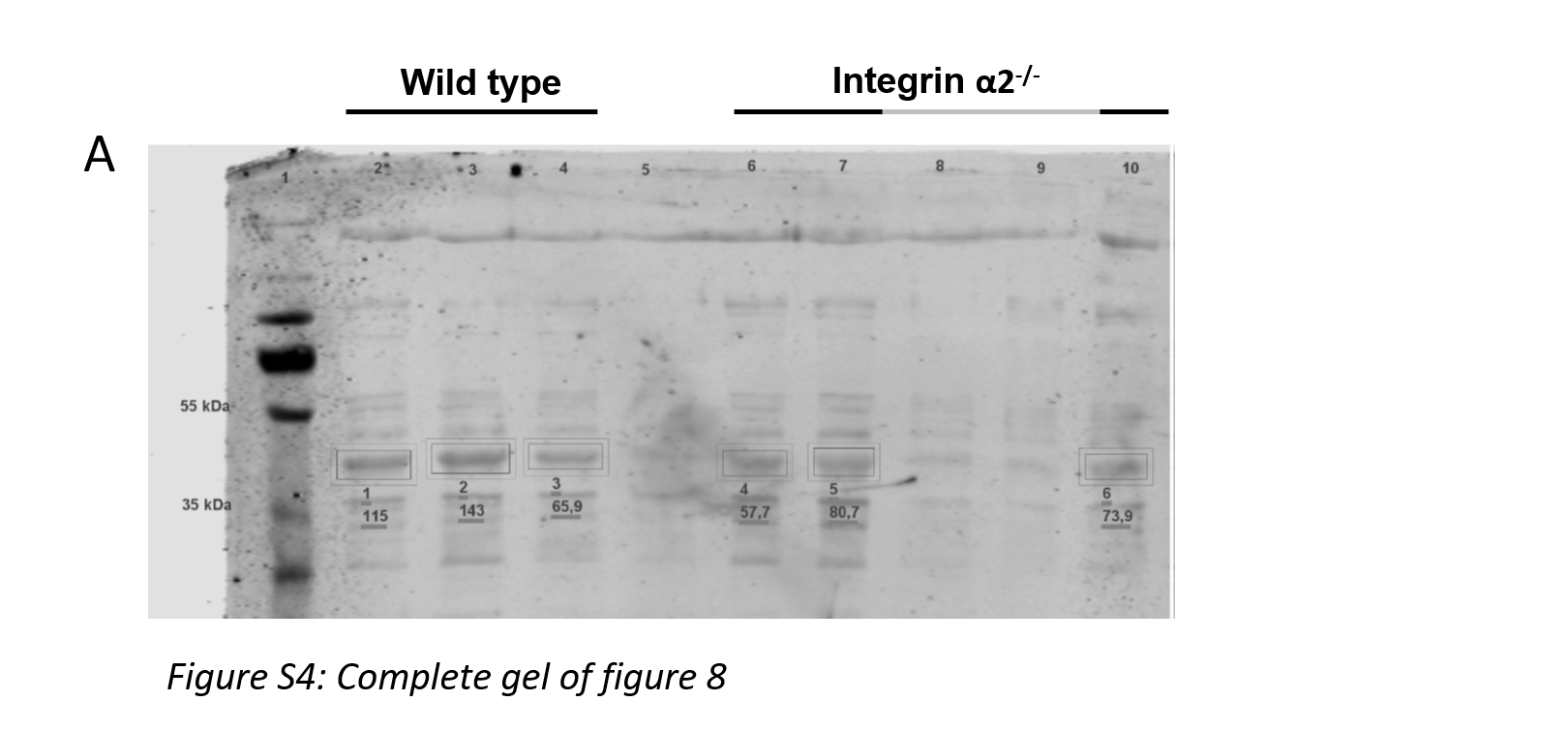

Supplement: Supplementary file 1 [file ijms-21-02835-s001.zip › Fig S4.tif]

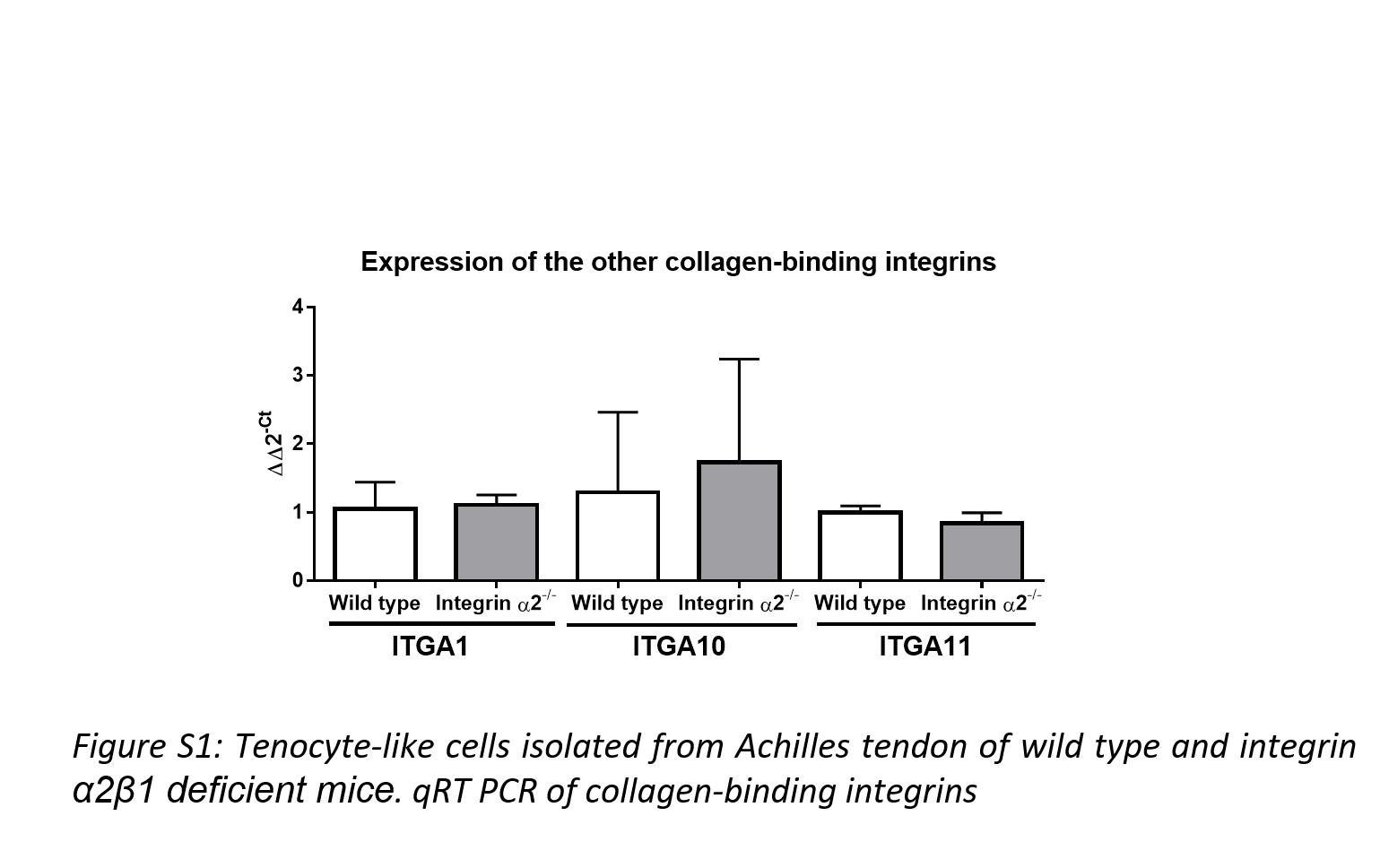

Supplement: Supplementary file 1 [file ijms-21-02835-s001.zip › Fig S1.tif]
